# A 6-week coordinative motor training program improves spatial ability performances in healthy children

**DOI:** 10.3389/fcogn.2024.1396399

**Published:** 2024-05-27

**Authors:** Christina Morawietz, Anna Maria Wissmann, Thomas Muehlbauer

**Affiliations:** Division of Movement and Training Sciences/Biomechanics of Sport, University of Duisburg-Essen, Essen, Germany

**Keywords:** intervention, physical exercise, visual-spatial abilities, orientation, movement coordination, youth

## Abstract

**Background:**

With overall academic achievements decreasing, policies tend to dedicate more curricular time to other subjects than physical education (PE). In light of increasingly sedentary lifestyles and rises in levels of overweight and obesity, this trend is detrimental within the global health context. Simultaneously, research on the connection between physical activity, cognitive functions, and academic achievement is on the rise. Cognitive functions like good spatial abilities have frequently been associated with higher achievements in STEM-subjects. This study is aimed to investigate the effects of a 6-week coordinative motor training with spatial elements on spatial ability performances in healthy children.

**Methods:**

Fifty-three children (mean age ± *SD*; 11.3 ± 0.6 years; 30 girls) participated in either a 6-week coordinative motor training (i.e., intervention group; 2x/week, 45 min/session) or attended regular PE class using the same volume (i.e., control group). Spatial abilities before and after the intervention period were evaluated in both groups using the Paper Folding Test (PFT), Mental Rotation Test (MRT), Water Level Task (WLT), Corsi Block Test (CBT), and Numbered Cones Run (NCR).

**Results:**

No significant differences between groups were observed at baseline. A main effect of test but not of group was found for all variables. For all but one test (i.e., PFT), a significant test × group interaction was detected. *Post-hoc* analyses revealed significant medium- to large-sized improvements from pre- to posttest in the intervention but not in the control group.

**Conclusion:**

The results indicate that a 6-week coordinative motor training with spatial elements is feasible in school-aged children and positively affects their spatial abilities.

## Introduction

The recent OECD PISA-study 2022 comes to the conclusion that overall achievements in core competencies like mathematics, reading, and science are decreasing (OECD, 2023). This fuels the trend to dedicate more curricular time to academic subjects rather than physical education (PE; Hardman et al., 2014; Doherty and Forés Miravalles, 2019; Youth Sport Trust, 2022).

Considering the increasingly sedentary lifestyles and physical inactivity in today's society, global health problems like overweight and obesity, mental health problems as well as non-communicable diseases like cardiovascular disease or type-2 diabetes are on the rise throughout all age groups (World Health Organisation, 2022; Phelps et al., 2024). Moreover, children's general motor abilities like muscular strength, endurance, flexibility, agility, or coordination are decreasing (Bös and Ulmer, 2003; Tomkinson et al., 2019; Masanovic et al., 2020). Child-care facilities and schools are key environments to bring children into contact with and facilitate an active and healthy lifestyle from an early age on (World Health Organization, 2021). School and PE-classes are the only point of contact with physical exercise for many children nowadays. Regular, high-quality PE-classes as well as any other form of physical activity (e.g., active breaks, active commute to and from school or activities before and after class) are therefore paramount within the educational context (Woods et al., 2021; World Health Organization, 2021). Yet, barriers for PE-classes are rising due to policies, less curricular time, poor student participation, lack of (qualified) teachers as well as insufficient or lacking equipment and facilities (Institute of Medicine, 2013; Hardman et al., 2014).

At the same time, the association between motor skills, physical activity or fitness and cognitive function in children and adolescents is receiving increasing attention in the research community in recent years (e.g., Chaddock-Heyman et al., 2018, 2020; Jia et al., 2021; Wick et al., 2022). The idea that motor and cognitive development are closely related goes back to early developmental theories (Frick and Möhring, 2015). Already very small children interact with their environment and explore their surroundings as soon as increasing motor abilities enable more motor independence (Frick and Möhring, 2015; Farran et al., 2019). This aids cognitive development and generates a basis for the development of spatial knowledge and spatial perception (Campos et al., 2000; Frick and Möhring, 2015; Farran et al., 2019). This idea is supported by a variety of studies. Davis et al. (2011) for example, found that cognitive and motor skills are closely interrelated in healthy 4–11-year-old children as measured by standardized tests, which indicates a developmental link. Similarly, Diamond (2000) concludes that cognitive and motor development are linked more closely than expected and perturbations (e.g., developmental disorders) tend to affect not only one, but both systems. A systematic review by van der Fels et al. (2015) further finds weak-to-strong correlations for complex motor skills and higher order cognitive skills in 4–16-year-olds with typical development. These findings indicate that complex motor interventions are suitable to train not only motor skills but also higher order cognitive skills.

Even though the overall evidence base is still scarce and more high-quality research is needed (van der Fels et al., 2015; Donnelly et al., 2016; Bidzan-Bluma and Lipowska, 2018; Singh et al., 2018), there are promising findings supporting the relation between physical activity, cardiorespiratory fitness and cognitive function (Donnelly et al., 2016; Alvarez-Bueno et al., 2020). In relation to academic achievements, there is evidence that higher levels of physical fitness in children correlate with superior performance in mathematics, but there are also benefits for reading and language performance (Chaddock-Heyman et al., 2015; Alvarez-Bueno et al., 2020). Research further suggests that increases in physical activity positively affect memory, attentional control, as well as behavior within the classroom (for reviews see Sibley and Etnier, 2003; Trudeau and Shephard, 2008). Particularly children before puberty seem to be susceptive for these enhancements (van der Fels et al., 2015).

In accordance with the idea that spatial knowledge and perception develop with increasing motor skills (Farran et al., 2019), a recent systematic scoping review reports emerging evidence of specific physical exercise interventions facilitating spatial competencies in children and adolescents (Morawietz and Muehlbauer, 2021). There is evidence that particularly interventions involving high levels of motor coordination, appear to have a positive impact on these complex cognitive functions (e.g., Jansen et al., 2011; Blüchel et al., 2013; Dirksen et al., 2015; Pietsch et al., 2017; Boraczyński, 2019; Latino et al., 2021). These findings are in line with van der Fels et al. (2015) realization that complex cognitive skills can be improved with complex motor interventions. Additional evidence from Moreau et al. (2012), Voyer and Jansen (2017), and Jansen and Pietsch (2022) indicates that specialization in sports and motor expertise positively influence specific cognitive functions like spatial abilities.

Spatial abilities are an aspect of cognitive function that is frequently associated with gaining independence and autonomy while growing up. In everyday life, spatial abilities are encountered when reading a map, finding the way, orienting oneself in an unknown environment, following and understanding directions, understanding spatial relations, information, shapes and patterns or solving problems (Tzuriel and Egozi, 2010; Fernandez-Baizan et al., 2019). Good spatial abilities have further been associated with higher academic achievements. Especially STEM-subjects (science, technology, engineering, and mathematics) appear to benefit from good spatial competencies and promote occupational success in this field (National Research Council, 2006; Uttal et al., 2013; Ishikawa and Newcombe, 2021). Moreover, good spatial skills are essential to successfully participate in team sports like football, gymnastics, or combat sports (Heppe et al., 2016; Voyer and Jansen, 2017).

These findings emphasize the importance of encouraging and facilitating the development of spatial abilities from an early age on. To date, teaching spatial competencies is not part of the regular school-curriculum yet (National Research Council, 2006; Wai et al., 2009). Moreover, attempts to enhance children's spatial abilities are mainly based on measures like traditional paper-and-pencil tasks, block building activities or computer-based interventions (Casey et al., 2008; Hawes et al., 2015; Lowrie et al., 2018). It is therefore of interest to continue to explore alternative ways to promote these cognitive skills.

Several systematic reviews conclude that a reallocation of curricular time toward PE and physical activity does not have negative influence on academic achievement (e.g., Trudeau and Shephard, 2008; Donnelly et al., 2016). Therefore, more research should take advantage of the preliminary findings regarding the positive interaction between physical activity and cognitive skills like spatial abilities. High-quality intervention studies are needed to pursue this line of evidence as this research field could open new possibilities to address several problems of today's society at once.

Given the information above, the present study aimed to investigate the effects of a 6-week coordinative motor training with spatial elements on spatial ability performances in healthy children. Based on previous research (Blüchel et al., 2013; Pietsch et al., 2017; Latino et al., 2021), we hypothesized that the specific intervention program would enhance spatial ability performances in healthy children compared to those attending regular PE classes.

## Methods

### Participants

Fifty-three healthy children (30 females and 23 males; age: 11.3 ± 0.6 years; body height: 161.1 ± 7.7 cm; body mass: 57.6 ± 14 kg; body mass index: 22.1 ± 4.7 kg/m^2^) voluntarily participated in this study. All participants attended the sixth grade of a public secondary school in the Ruhr area of North Rhine-Westphalia, Germany. Subjects were unaware of the training protocol and had no previous experience with the implemented set of tests. All participants and their legal guardians provided informed consent before entering the study. The study protocol was approved by the Human Ethics Committee of the University of Duisburg-Essen, Faculty of Educational Sciences (EA-PSY20/23/04102023).

Using G^*^Power (version 3.1.9.7; Faul et al., 2007) the power analysis (*f* = 0.25, α = 0.05, 1-β = 0.80, number of groups: *n* = 2, number of measurements: *n* = 2, correlation between testing: *r* = 0.30, drop-out rate per group: 10% due to injury reasons not attributable to treatments) revealed that a total sample size of *N* = 50 participants would be sufficient to detect statistically significant treatment effects.

### Experimental design

**Figure 1:**
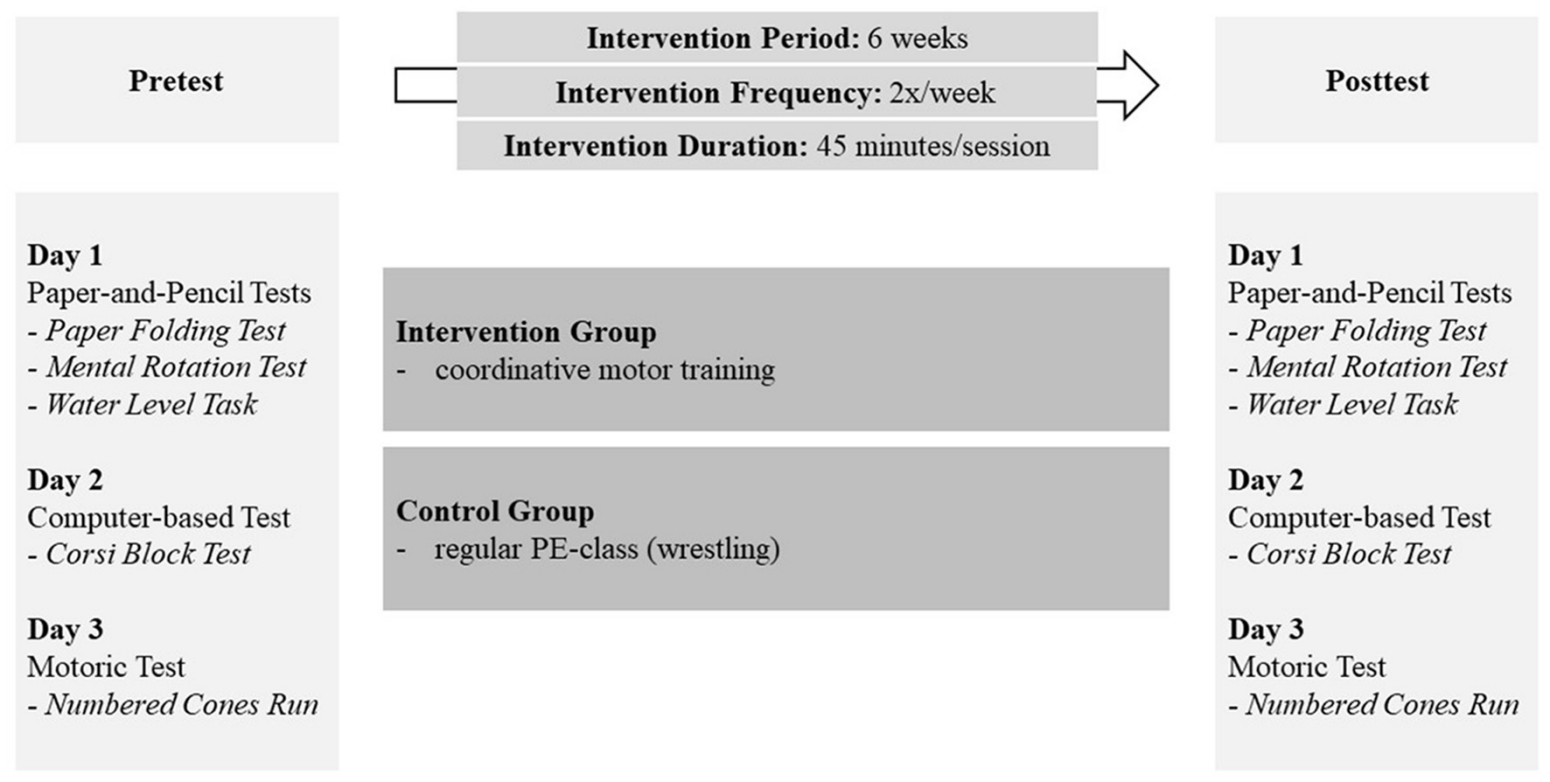
Schematic description of the study design.

The study was conducted from October to December 2023. As the investigation was performed within a school setting, group allocation on a class basis took place by lottery. One class of 27 pupils (12 females and 15 males) was therefore assigned to be the intervention group (INT) and performed a 45-min spatial ability intervention twice per week. The other class of 26 pupils (11 females and 15 males) was assigned to be the control group (CON) and attended regular PE class using the same volume. Prior to the intervention, a pretest was performed with all participants. The identical assessment was performed as posttest upon completion of the intervention period (Figure 1).

### Testing procedure

The pretest and posttest each took place on 3 testing days and covered a variety of spatial ability aspects. Standardized verbal instructions were provided prior to each assessment. The 1st testing day was executed in the students' classroom, where participants individually performed three paper-and-pencil assessments from a test-booklet. A visual demonstration of each test was performed during instructions. The *Paper Folding Test* (*PFT*; Ekstrom et al., 1976) was chosen as a measure of spatial visualization. The task consisted of the depiction of 20 sheets of paper that were folded in different ways, a hole was then punched through them, and the paper was unfolded again. For each item, participants had to select the correct answer out of five options. The PFT was divided into two parts with ten items each. Each part had a time-limit of 3 min separated by a 3-min break. The PFT included one practice item that had to be completed prior to the first part. A maximum of 20 points could be reached. To evaluate mental rotation, the *Mental Rotation Test (MRT) Version A* by Peters et al. (1995; based on Vandenberg and Kuse, 1978) was selected. A three-dimensional block figure was depicted as a target object. Out of four possible answers, participants had to decide which two showed rotated and tilted versions of the target object. Similar to the PFT, the MRT comprised of two parts with 12 items each. Each part had to be solved within a time-limit of 3 min with 3 min break in between. Four practice items with a time-limit of 5 min were completed in advance. One point was allocated if both answers were correct resulting in a maximum of 24 points. Lastly, the *Water Level Task* (*WLT*; Piaget et al., 1956) in the version by Yingying Yang (University of Alabama; Merrill et al., 2016) was employed to test spatial perception. Participants had to imagine how the water level would look like when 12 empty jars tilted to various degrees were half filled with water. They were then asked to draw the respective waterlines. The time-limit was 3 min. For lines within the tolerance range of ±10° from horizontal, participants received one point, amounting to a maximum of 12 points. Reliability of the PFT (ICC = 0.78), MRT (ICC = 0.81), and WLT (ICC = 0.88) has been shown in one of our previous studies (Morawietz et al., 2024).

Spatial learning, visuospatial short-term- and working memory were assessed on the 2nd testing day using a computerized version of the *Corsi Block Test* (*CBT*; Corsi, 1972). The self-programmed test was based on the online-demo of Millisecond Software, Seattle, USA and in line with the normative data provided by Kessels et al. (2000). While instructions took place in small groups of five students, the test was performed by each participant individually in a separate room. Nine blue squares were presented on a black laptop screen. In sequences of increasing length some of the squares lit up in yellow. Starting with two squares (block sequence one and two), one square was added to every subsequent sequence. Using a computer mouse, participants were asked to immediately repeat the sequences. At least one out of two trials per block sequence length had to be repeated correctly. Otherwise, the test was instantly terminated. The CBT was performed without time-limit and the block span (max. 9 points), and total score (max. 144 points) were calculated. The CBT (span: ICC = 0.95; composite score: ICC = 0.93) proved to be reliable in one of our previous studies (Morawietz et al., 2024).

Evaluating spatial orientation, an adapted version of the Medicine Ball Number Run (Jung, 1983 as cited in Hirtz et al., 2010), the *Numbered Cones Run (NCR)*, was performed on the 3rd day. Instruction and assessment took place in small groups of five students. Five cones were randomly numbered and set up in a semicircle with 1.5-m distance from each other. The starting point was marked at 3-m distance. Standing with their back to the numbered cones, a number was called out to the participants, and they were asked to run to the respective cone as fast as possible, touch it and return to the starting point. Upon return, the procedure was repeated immediately for a second and third time. The running order was predetermined by a random number generator and differed between participants. Reliability of the NCR (ICC = 0.91) has been shown in one of our previous studies (Morawietz et al., 2024).

### Training procedures

The training took place during regular PE class over a period of 6 weeks (twice per week). Each PE class lasted 60 min and included a 5–10-min warm-up and 30–35 min of exercise. Fifteen minutes were allocated to changing and setting up/taking down of equipment. While the CON followed the regularly scheduled curriculum with wrestling exercises, the INT performed a specifically designed training program focusing on motor coordination with spatial ability elements (Table 1). Training of the INT was performed by a graduated sport scientist. The training program consisted of 12 self-contained sessions and was conceptualized on the basis of various intervention studies in the field of spatial abilities (e.g., Jansen et al., 2011, 2013; Blüchel et al., 2013; Dirksen et al., 2015; Pietsch et al., 2017; Boraczyński, 2019; Latino et al., 2021). Training sessions included Le Parkour inspired obstacle courses; obstacle courses that needed to be completed as a team; stations with coordination exercises; ball coordination exercises including throwing and catching; juggling; football and volleyball coordination; dancing and coordination games. Possibilities for progression were offered and encouraged for all tasks applicable.

**Table 1:** Description of the training procedures by group.

**Session**	**Intervention group**		**Control group**	
	**Description**		**Description**	
	**Duration (min)**	**Content**	**Duration (min)**	**Content**
1	5–10	**Warm-up game** (i.e., fire, water, lightning: participants run and perform e.g., jumps, planks and turns with add. orientation component [e.g., fw, bw, and sw] on command)	5–10	**Warm-up game** [i.e., greeting: participants move freely through the gym and, once the music stops, greet each other with a part of the body called out (e.g., touch as many feet as possible with one's own foot, knees with one's knee)]
	30–35	**Movement parcours** (e.g., participants jump from small to large to small gymnastics box; participants jump from gymnastic box onto horizontal bar and then onto a mat; participants swing between horizontal bars)	30–35	**Getting closer** (i.e., first physical contact games to promote and improve interaction between participants)
		*Progressions:* e.g., increasing distances, double/single leg jumps/landings, additional turns, adding run-ups, balancing on bar		
2	5–10	**Warm-up game** (i.e., line-tag: participants are only allowed to run on lines displayed on gym floor and have to remain on their spot as additional barriers, once they are caught)	5–10	**Warm-up game** (i.e., fire, water, lightning: participants run and perform e.g., jumps, squats and push-ups on command
	30–35	**Coordination stations** (e.g., participants balance on borders of vaulting boxes; participants try to walk on a line with closed eyes after turning in circles; in pairs: each partner balances on a bench sw while balancing a ball on two sticks together)	30–35	**Falling safely—Part 1** (i.e., participants learn playfully how to fall safely in various movement situations)
		*Progressions:* e.g., direction changes, open/closed eyes, crossing arms/legs		
3	5–10	**Warm-up game** (i.e., dribbling-tag: participants each dribble a ball and a catcher tries to hit a ball away to “catch”)	5–10	**Warm-up game** (i.e., sandwich-game: participants move freely through the gym, fast-foods are called and participants have to lie on top of each other in correspondingly large groups [e.g., sandwich: two, cheeseburger: four], left over participants can re-join the game after performing ten jumping jacks)
	30–35	**Ball coordination** (**throwing and catching**; e.g., participants throw a ball up in the air, roll forward and catch the ball again; two participants stand behind each other with the back person throwing a ball against a wall and the front person catching the ball when it bounces back; participants stand in a square in groups of four and passing two balls)	30–35	**Falling safely—Part 2** (i.e., participants learn playfully how to fall safely in various movement situations and improve the movements they have already learned)
		*Progressions:* e.g., adding (several) turns/rolls, additional balls, kicking one ball and throwing the other		
4	5–10	**Warm-up game** (i.e., juggling-tag: several catchers, the other participants have three juggling balls that are passed between them; if participants are caught, they can be freed again by catching a juggling ball that is thrown over their shoulder and passing it back through their legs	5–10	**Warm-up game** (i.e., high tide: participants move freely through the gym, on command they have to save themselves onto islands [e.g., vaulting boxes, benches, mats]; islands are gradually reduced, participants have to help each other)
	30–35	**Juggling** (e.g., participants throw one ball up, clap their hands and catch the ball again; participants throw two balls up, cross their arms and catch the balls again; cascade)	30–35	**Fighting according to rules and rituals** (i.e., development of rules and rituals through a variety of exercises to test strength)
		*Progressions:* e.g., adding additional balls		
5	5–10	**Warm-up game** (i.e., hoop-relay in two groups: each participant has to run through eight hoops as fast as possible before the next group member starts)	5–10	**Warm-up game** (i.e., ribbon-tag: each participant has a ribbon fastened to their pants; the catcher tries to steal a ribbon; participant the ribbon is stolen from becomes new catcher
				*Variation:* several catchers
	30–35	**Le Parkour** (e.g., participants perform precision jumps from one gymnastic bench to the next; participants jump and roll between horizontal bars without touching them; participants perform wallruns)	30–35	**Measure strength** (i.e., repetition and consolidation of rules through strength measurement exercises and ball fights with partners at stations and in a games)
	**Description**		**Description**	
	**Duration (min)**	**Content**	**Duration (min)**	**Content**
		*Progressions:* e.g., adding turns, single leg jumping/landing, additional steps on the wall		
6	5–10	**Warm-up game** (i.e., animal-relay in small groups: participants have to move like animals in predetermined ways)	5–10	**Warm-up game** [i.e., two feet, three hands: participants have to build figures in groups of three where only a predetermined amount of body parts can touch the floor (e.g., two feet and three hands)]
				*Variation:* groups have to move a certain distance in this position
	30–35	**Coordination games** (e.g., orientation-relay with three teams: cones in different colors are spread throughout the gym, participants have to run to each cone of their color and surround it before moving to the next; atom-game: participants move freely through the gym, numbers are called and participants have to meet in correspondingly large groups, left over participants can re-join the game after performing 10 jumping jacks)	30–35	**Getting to know partner fights** (i.e., development and testing out of different partner fighting games using picture cards)
		*Progressions:* in each group, certain amounts of certain extremities can/have to touch the floor, i.e., three feet and two hands in a group of four		
		*Variations:* e.g., all participants are blindfolded		
7	5–10	**Warm-up** (i.e., each participant dribbles a football, once the music stops, balls have to be passed between participants)	5–10	**Warm-up game** (i.e., animal-relay in small groups: participants have to move like animals in predetermined ways)
	30–35	**Ball coordination** (**football**; e.g., in teams of three: participants pass a ball over a bench, then run around the bench to pass back again; participants take shots at cones on a bench; all participants simultaneously dribble through several slalom-courses without getting in each other's way)	30–35	**Practice at combat training stations** (i.e., participants try out and invent wrestling and combat exercises at various stations to deepen their awareness of the rules)
		*Progressions:* e.g., using the other foot		
8	5–10	**Warm-up** (i.e., around the world: participants run through the gym imitating airplanes, when the music stops, they perform dances of different countries)	5–10	**Warm-up game** [i.e., greeting: participants move freely through the gym and, once the music stops, greet each other with a part of the body called out (e.g., touch as many shoulders as possible with one's own shoulder, ears with one's ear)]
	30–35	**Creative dance** (dance-story: parcours representing a story where participants have to e.g., jump through strawberry fields, roll under a bridge, slalom through stinging nettles, jump over a river)	30–35	**Practice at combat training stations** (i.e., participants try out and invent wrestling and combat exercises at various stations to deepen their awareness of the rules)
		*Variations:* e.g., slow-motion, fast, bw, and with different emotions		
9	5–10	**Warm-up game** (i.e., fire, water, lightning and storm: e.g., benches, mats, and vaulting boxes are built up in the gym, participants run around and perform tasks like lying down, moving onto an obstacle or holding onto sth. on command)	5–10	**Warm-up game** [i.e., pirates and nobles: two teams positioned on each side of the gym, nobles have various treasures behind them (e.g., balls and ribbons) and pirates have to try to steal those objects and bring them to their cave; on the way back, treasures can be recaptures by nobles; treasures cannot be thrown]
	30–35	**Coordination stations** (in groups of four: e.g., a treasure-hunt: one person is blindfolded, while the other person tries to guide to a hidden object with acoustic instructions, each team tries to be faster than the other team; two participants simultaneously jump rope with only one jump rope; blindfold slalom: participants try to remember a slalom course and walk it blindfolded)	30–35	**Practice at combat training stations** (i.e., participants try out and invent wrestling and combat exercises at various stations to deepen their awareness of the rules)
		*Progressions:* e.g., increasing speed		
10	5–10	**Warm-up game** (i.e., two teams: each team tries to pass a ball seven times between members to score a point, the other team tries to interrupt and score themselves)	5–10	**Warm-up game** [i.e., catch and release: participants play tag; caught participants can be released again by predetermined actions (e.g., crawling through spread legs, high-five combination with both hands/one hand hugging)]
	30–35	**Ball coordination** (e.g., in groups of two with three balls: one participant throws a ball up and passes the other ball to the partner before catching the first ball again, back and forth without breaks; in groups of two: one person stands with the back to the partner, who bounces a ball through the frame of a vaulting box. On command, the first person has to turn and catch the ball; in teams of four with two balls: one ball has to be thrown and caught with the hand, the other ball can be played with any part of the body except the hand, participants pass the ball between team members and change position after each ball contact)	30–35	**Aptitude test** (i.e., presentation, testing, and securing of participants' own combat exercises)
		*Progressions:* e.g., additional ball that has to be rolled, additional turns, and bouncing several times		
11	5–10	**Warm-up game** (i.e., fire water, lightning and storm: see session 9)	5–10	**Warm-up game** (i.e., high tide: see session 4)
	30–35	**“Mount Everest”—obstacle course** (i.e., obstacle round course with e.g., benches, mats, horizontal bars, vaulting boxes, vault, balance beam, and uneven bars where participants have to “climb Mount Everest” in teams of two without falling)	30–35	**Final tournament—Part 1** (i.e., fights in small groups between participants in accordance with the established rules and rituals)
		*Progressions:* e.g., one partner supports from ground, partners have to help each while both are in the obstacle course, change of direction, passing other teams that are approaching in the other direction, carrying a ball		
12	5–10	**Warm-up game** (i.e., cross-tag: chased participants can save themselves by touching another participant who then becomes catcher as well)	5–10	**Warm-up game** (i.e., two feet, three hands: see session 6)
	30–35	**Coordination games** [e.g., guard game: participants try to place balls in two boxes, four guards try to prevent this and try to eliminate players from game by stealing a ribbon fastened to their pants; place swap: two teams, each team passes a ball in a predetermined way (e.g., clockwise), once participants pass the ball, they have to swap place with the person opposite to them]	30–35	**Final tournament—Part 2** (i.e., fights in small groups between participants in accordance with the established rules)
		*Progressions:* additional tasks, walking bw		

bw, backwards; fw, forwards; sw, sideways.

### Statistical analysis

Prior to the conduction of parametric analyses, normal distribution (Shapiro–Wilk Test) and variance homogeneity (Mauchly Test) were checked and confirmed. Data were presented as group mean value ± standard deviation (*SD*). Afterwards, a series of 2 (test: pretest, posttest) × 2 (group: intervention, control) repeated measures ANOVA were performed. If a significant test by group interaction occurred, Bonferroni-adjusted *post-hoc* tests (i.e., paired *t*-test) were applied. The significance level was *a priori* set at α <0.05. For the ANOVA, effect size was calculated as partial eta-squared ($\eta_{\text{p}}^{2}$) and reported as small (0.02 ≤ $\eta_{\text{p}}^{2}$ ≤ 0.12), medium (0.13 ≤ $\eta_{\text{p}}^{2}$ ≤ 0.25), or large ($\eta_{\text{p}}^{2}$ ≥ 0.26; Cohen, 1988). For the paired *t*-test, effect size was calculated as Cohen's *d* and stated as small (0 ≤ *d* < 0.50), medium (0.50 ≤ *d* < 0.80), or large (*d* ≥ 0.80; Cohen, 1988). All analyses were performed using SPSS version 28.0 (IBM Inc., Chicago, IL).

## Results

**Figure 2:**
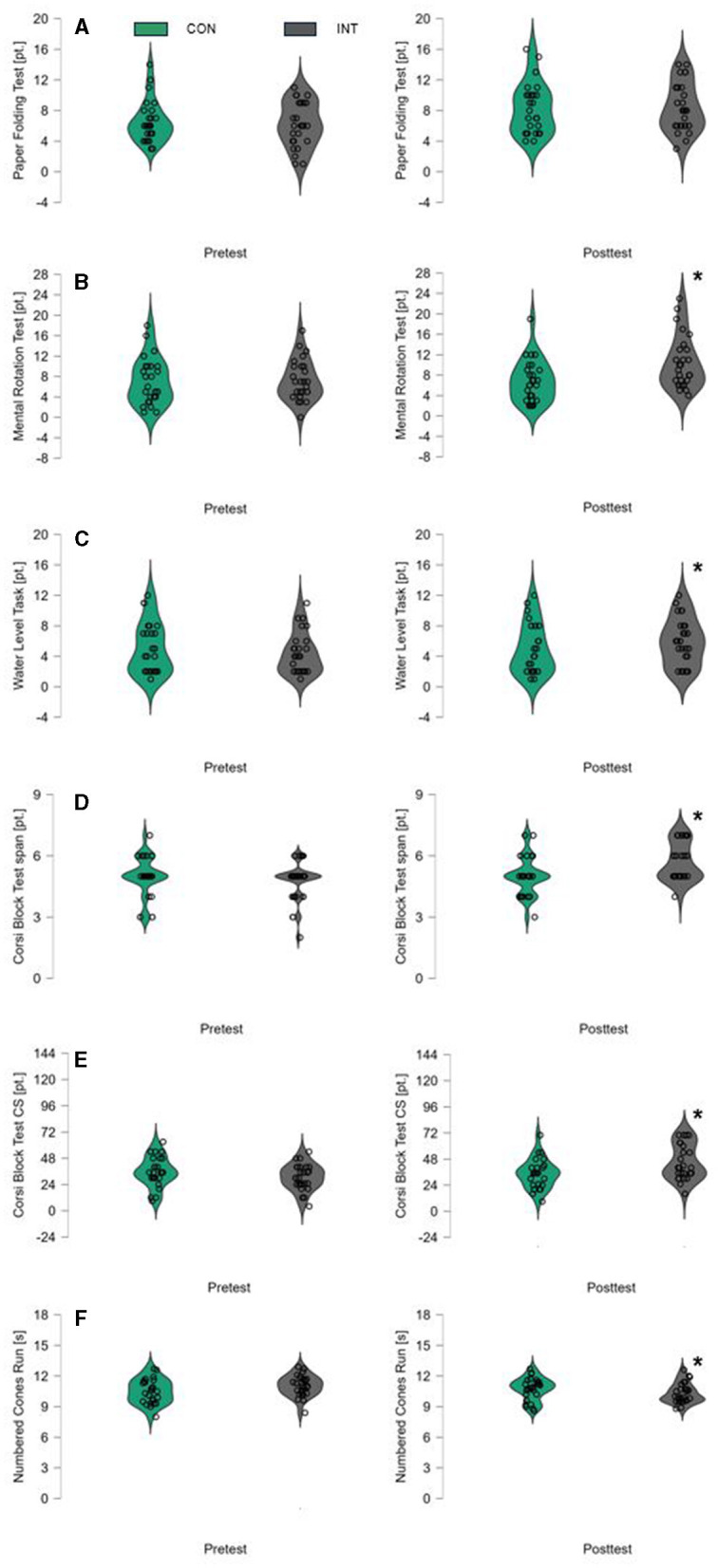
Violin plots showing performance changes from the pretest to the posttest in the Paper Folding Test **(A)**, the Mental Rotation Test **(B)**, the Water Level Task **(C)**, the Corsi Block Test span **(D)**, the Corsi Block Test composite score **(E)**, and the Numbered Cones Run **(F)** for the control (green) compared to the intervention (gray) group. *Represents a statistically significant difference to the pretest (*p* < 0.001). CON, control group; CS, composite score; INT, intervention group.

Table 2 displays descriptive statistics and Table 3 displays inference statistics for all analyzed variables. Generally, there were no statistically significant differences in pretest values between both groups. Further, the attendance rates during training sessions amounted to 95.7 and 94.6% in the INT and CON, respectively. For all variables, the statistical analysis showed a significant main effect of test but not of group. Further, a significant test × group interaction was detected for all but one (i.e., PFT) measure. *Post-hoc* analyses revealed significant medium- to large-sized improvements from the pretest to the posttest in the INT (MRT: *p* < 0.001, Cohen's *d* = 0.66; WLT: *p* < 0.001, Cohen's *d* = 0.51; CBT-span: *p* < 0.001, Cohen's *d* = 0.99; CBT-CS: *p* < 0.001, Cohen's *d* = 1.00; NCR: *p* < 0.001, Cohen's *d* = 0.76) but not in the CON (MRT: *p* = 0.355, Cohen's *d* = 0.04; WLT: *p* = 0.361, Cohen's *d* = 0.02; CBT-span: *p* = 0.500, Cohen's *d* = 0.01; CBT-CS: *p* = 0.348, Cohen's *d* = 0.08; NCR: *p* = 0.075, Cohen's *d* = 0.19; Figures 2A–F). Additionally performed gender-specific sub-analysis did not reveal any significant effects (data not shown).

**Table 2:** Effects of a 6-week coordinative motor training on measures of spatial ability performance in healthy children by group.

**Outcome**	**Intervention group (*****n*** = **27)**		**Control group (*****n*** = **26)**	
	**Pretest**	**Posttest**	**Pretest**	**Posttest**
Paper Folding Test [pt.]	6.2 ± 2.9	8.4 ± 3.2	6.5 ± 2.7	8.2 ± 3.3
Mental Rotation Test [pt.]	7.2 ± 3.9	10.4 ± 5.1	7.0 ± 4.5	6.9 ± 4.1
Water Level Task [pt.]	4.3 ± 2.7	5.9 ± 3.1	5.0 ± 3.3	4.9 ± 3.3
Corsi Block Test span [pt.]	4.7 ± 0.9	5.7 ± 0.9	5.0 ± 1.0	5.0 ± 0.9
Corsi Block Test CS [pt.]	30.8 ± 11.7	45.4 ± 16.4	35.6 ± 13.6	34.6 ± 13.4
Numbered Cones Run [s]	11.0 ± 1.0	10.3 ± 1.0	10.5 ± 1.2	10.7 ± 1.1

Values are means ± standard deviations.

**Table 3:** Main and interaction effects of the repeated measures ANOVA for each outcome measure.

**Outcome**	**Main effect: test**		**Interaction effect: test** × **group**	
	*F*_(df)_	***p*** **(**$\eta_{\text{p}}^{2}$**)**	*F*_(df)_	***p*** **(**$\eta_{\text{p}}^{2}$**)**
Paper Folding Test [pt.]	_(1, 51)_ = 24.955	<0.001 (0.33)	_(1, 104)_ = 0.422	0.519 (0.01)
Mental Rotation Test [pt.]	_(1, 51)_ = 14.954	<0.001 (0.23)	_(1, 104)_ = 18.991	**<0.001 (0.27)**
Water Level Task [pt.]	_(1, 51)_ = 15.162	<0.001 (0.23)	_(1, 104)_ = 18.571	**<0.001 (0.27)**
Corsi Block Test span [pt.]	_(1, 51)_ = 10.011	0.003 (0.16)	_(1, 104)_ = 10.011	**0.003 (0.16)**
Corsi Block Test CS [pt.]	_(1, 51)_ = 12.51	<0.001 (0.20)	_(1, 104)_ = 16.639	**<0.001 (0.25)**
Numbered Cones Run [s]	_(1, 51)_ = 4.582	0.037 (0.08)	_(1, 104)_ = 15.673	**<0.001 (0.24)**

The bold values indicate statistically significant interaction effects (p <0.05). Threshold values for the $\eta_{\text{p}}^{2}$-value were 0.02 ≤ $\eta_{\text{p}}^{2}$ ≤ 0.12 = small, 0.13 ≤ $\eta_{\text{p}}^{2}$ ≤ 0.25 = medium, and $\eta_{\text{p}}^{2}$ ≥ 0.26 = large. CS, composite score.

## Discussion

The present study evaluated the effect of a 6-week coordinative motor training with spatial elements on spatial ability performance in healthy children. In line with our hypothesis, children in the INT improved significantly from pre- to posttest in four out of five spatial ability measures compared to the CON.

### Effects on spatial abilities

Our findings are supported by previous research evaluating the effect of motoric interventions on spatial abilities in children and adolescents. Mental rotation performance of the INT increased from 7.2 to 10.4 points (group mean values) from pre- to posttest but remained stable in the CON (pretest: 7.0 points, posttest 6.9 points) indicating that the intervention positively affected mental rotation performance of the INT. Blüchel et al. (2013) for example, report that mental rotation performance in children (mean age ± *SD*: 9.06 ± 0.45 years) improved significantly after 2 weeks of specific coordinative motor training including throwing, catching and bouncing tasks, skateboarding or motor memory games compared to children attending regular classroom-based lessons. The MRT and the test conduction was identical to the one used in the present study. Similar results are disclosed by Jansen et al. (2013) who recorded improvements in mental rotation performance of children (mean age ± *SD*: 7.68 ± 0.503 years) after 5 weeks of creative dance training as well as Pietsch et al. (2017) who found positive effects after performing a Life Kinetik-motion program with cognitive, coordinative, and visual task complexes respectively with children (mean age ± *SD*: 8.65 ± 0.482 years). Control groups in both studies attended regular PE-class and child-adapted MRTs were applied with animals or letters as stimuli.

Similar findings were observed for the WLT. While the INT increased their group mean values from 4.3 to 5.9 points between pre- and posttest, slight decreases were observed in the CON (pretest: 5.9 points, posttest: 4.9 points). Even though the test has been extensively researched over the past decades, to our knowledge no other motoric intervention studies made use of the WLT as measure of spatial abilities impeding the discussion of our findings based on previous literature. Various developmental stages of problem-solving have been proposed by Piaget et al. (1956) which are associated with the age of the participants. Their approach has been refuted over the years because even though all strategies appear to be used by children when solving the task, they appear to be used independent of age and switches between strategies occur (Thomas et al., 1993). Thomas et al. (1993) discuss three different response strategies (i.e., a bottom parallel approach, a random-like approach, and a correct approach) which are commonly seen in children and adolescents. While younger children tend to use a bottom parallel approach and the correct understanding tends to develop with increasing age, shifts between strategies and influential factors like the degree of inclination of the vessel or field effects also appear to affect responses (Thomas et al., 1993; Lohaus et al., 1996; Vasta and Liben, 1996). This does not only account for children but also for adults. Moreover, knowledge and understanding of the principle of horizontality of liquids appears to be inevitable to solve the WLT (Hecht and Proffitt, 1995). Hecht and Proffitt (1995) further established that adoption of an allocentric frame of reference is required to master the task successfully. This finding is supported by Barhorst-Cates et al. (2020), who report that children (mean age ± *SD*: 9.28 ± 0.76 years) who are able to adopt environmental views when asked to draw a map of their home environment tend to perform better on the WLT than children who adopt an egocentric perspective. Looking at our results, only few participants managed to solve the majority of items correctly and deviations of more than 50° occurred at pre- and posttest. Still spatial perception [i.e., spatial relations in regard of the own body's position as defined by Linn and Petersen (1985)] or extrinsic-static spatial skills [i.e., the spatial relation of an object in context of other objects or the environment according to the categorization of spatial abilities by Newcombe and Shipley (2015)] as measured by the WLT appear to be addressed and promoted by our intervention.

The INT also improved their performance on the CBT. The span as well as the CS increased from 4.7 points (pretest) to 5.7 points (posttest) and from 30.8 points (pretest) to 45.4 points (posttest), respectively. In contrast, values of the CON remained stable or even decreased slightly from pretest (span: 5.0 points; CS: 35.6 points) to posttest (span: 5.0 points; CS: 34.6 points). Comparable findings were obtained by Latino et al. (2021) who had adolescents (mean age ± *SD*: 14.4 ± 0.5 years) perform a 12-week coordinative ability training designed to improve cognitive skills. Performance on the CBT increased significantly from pre- to posttest in the INT but not in the CON, who participated in general psycho-physical wellness program. Similarly, Notarnicola et al. (2012) found significant differences between groups on the CBT forward and backward in favor of the INT after 26 weeks of orienteering lessons and orienteering exercises compared to the jogging CON. Moreover, the INT improved significantly from pre- to posttest on the backward CBT. Participant's mean age in this study was 9 years. Supported by these previous studies, our findings point toward an enhancement of visual-spatial working memory function based on the intervention.

Finally, results on the NCR revealed significantly faster completion times for the INT when looking at pre- and posttest (11.0 and 10.3 s, respectively), indicating positive effects of the motor-coordination intervention on spatial orientation. The CON on the other hand was not able to maintain or improve their performance (pretest: 10.5 s; posttest: 10.7 s). To our knowledge, the NCR is not widely used in the current research community. A study by Dirksen et al. (2015) found a significant main effect of time but not of group and no interaction effects when evaluating the results of 20 weeks of movement-coordination exercises during PE class compared to participation in regular PE class in children (mean age ± *SD*: 12 ± 0.46 years). Even though training content and participant age are similar the results of the study by Dirksen et al. (2015) and the results of the present study differ notably. This could be due to the fact that the training modalities [15 min/session, 2x/week for 20 weeks in Dirksen et al. (2015) vs. 45 min/session, 2x/week for 6 weeks in the present study] as well as training intensity and levels of load differed. Moreover, in contrast to the present study, *post-hoc* power analysis revealed that the sample was too small (*n* = 91) to reveal significant interaction effects due to high drop-out rates (Dirksen et al., 2015).

Unexpectedly, the PFT only showed a main effect of test but no test × group interaction effects. There are several possible reasons for this. For one, a recent paper by Uttal et al. (2024) aptly summarizes the difficulties in relation to spatial testing. Disagreement about spatial constructs in general and about the question which tests evaluate which spatial constructs in particular are widespread in the research community (Uttal et al., 2013, 2024). Therefore, the possibility exists that the PFT does not measure spatial visualization as expected by us. Burte et al. (2019) for example, claim that frequently different strategies (i.e., not only spatial visualization) are used to solve the tasks of the PFT and that participants might switch between strategies depending on the test item. Moreover, Uttal et al. (2024) point out that many tests have been designed and are available for certain age groups only with tests lacking that can be used across age groups. According to the test's authors, the PFT is suited for students from grade nine to 16 (Ekstrom et al., 1976). We tried to overcome this issue by evaluating test-retest reliability and minimal detectable change (MDC_95%_; i.e., the clinically relevant effect between repeated measurements of one subject) in younger children (mean age ± *SD*: 11.4 ± 0.5 years) and adolescents (mean age ± *SD*: 12.5 ± 0.7 years) prior to administering the test in the present study (Morawietz et al., 2024). Results indicated that the PFT would be suitable for this age group (ICC = 0.78), however, the test might not be sensitive enough to capture changes in the present age group or our sample might have been too small and not representative. Other spatial ability tests might have been more suitable to detect changes by our intervention. Another possible reason for the lack of improvement on the PFT is that spatial visualization might not have (sufficiently) been trained and affected by our intervention. Considering the categorization by Linn and Petersen (1985), spatial visualization involves complex processing of spatial information which might be executed in multiple steps or on multiple levels. According to the more recent categorization by Newcombe and Shipley (2015) the PFT can be assigned to the intrinsic-dynamic spatial skills, which involves the mental transformation and modification of the spatial information of objects (e.g., folding, rotation, or deformation). Even though contents like juggling and different ball coordination exercises should have addressed these skills, their frequency throughout the intervention period might have been too low. The small improvement from pre- to posttest in both groups can be attributed to learning and memory effects, due to repeated exposure to the same test stimuli. These learning effects have frequently been discussed in the literature before (e.g., Scharfen et al., 2018; Fehringer, 2023). Similar findings to ours have also been reported by Bakker (2008) who investigated the effect of the Tridio^®^ learning material (i.e., exercises with cubes and mosaics to enhance spatial abilities) in 5th-graders and did not find significant improvements in the INT compared to the CON.

### Implications within the school context

Our findings show that already a targeted intervention on a rather small scale (i.e., 6 weeks of training, twice per week for 45 min/session) can significantly facilitate spatial abilities in healthy children. Other research found that even shorter intervention periods (e.g., 2 weeks or 5 weeks of training) resulted in improvements of specific spatial skills (Blüchel et al., 2013; Pietsch et al., 2017). While schools have a strict curriculum for PE-classes like for any other subject, it would be worthwhile to integrate such small-scale interventions within the everyday school life or PE. The present intervention does not only affect spatial skills which in turn might impact on overall academic achievement but also addresses fundamental movement skills like the different aspects of motor coordination. As discussed earlier, these and other motor skills are increasingly declining in today's children and adolescents (Bös and Ulmer, 2003; Tomkinson et al., 2019; Masanovic et al., 2020). The diverse structure of the intervention program, the focus on participation rather than performance and the provision of individual variations in the level of difficulty for all tasks help to motivate students and allow the experience of success also for students that are less keen on sports. This is also backed by our results with attendance rates of 95.7 and 94.6% in the INT and CON, respectively. Moreover, such positive experiences might help in providing an access to a sports and movement culture in their leisure time for students that did not have this possibility beforehand. Therefore, integrating a coordinative motor training within the school context can contribute to tackle several current academic and health problems at once.

### Limitations and directions for future research

While the overall outcomes of this study are very promising, several limitations need to be taken into account. Even though our sample size was large enough to generate meaningful outcomes, the overall sample was rather small and there is a need for larger studies to provide a stronger base of evidence. Moreover, only healthy children of a specific age were investigated in the present study. Our results are therefore neither transferable to other age groups nor to participants with (cognitive) impairments. Future research should replicate the present study with other age groups to confirm our findings, to work out discrepancies and to facilitate knowledge about developmental steps with regard to spatial abilities. The same accounts for the spatial ability measures used. Findings do neither account for any other measure of spatial ability nor for any adaptation of the tests used not even if they might be assigned to the same category of spatial tests. As discussed earlier, there is disagreement about the constructs of spatial abilities in the research community with two commonly used and in parts overlapping categorizations being those of Linn and Petersen (1985; i.e., spatial perception, mental rotation, and spatial visualization) and Newcombe and Shipley (2015; i.e., intrinsic-static, intrinsic-dynamic, extrinsic-static, and extrinsic-dynamic; Uttal et al., 2013, 2024). Generally speaking, reliable and valid spatial ability tests appear to be lacking, many measures and/or answer keys are difficult to access or expensive and there are inconsistencies regarding the question which test is suitable to measure which spatial construct (Uttal et al., 2024). While some tests have names that sound similar, they actually measure very different constructs. On the other hand, there are tests that allegedly measure different constructs when in fact they evaluate the same spatial skills (Hegarty and Waller, 2005; Uttal et al., 2024). Even though extensively researched, the possibility exists, that the tests used in the present research do in fact measure different spatial constructs than we intended to, particularly as we used them in a different age group than most tests were developed for. Also, other spatial ability measures might have been more suitable and informative to evaluate our intervention. This might particularly be true for the NCR. Even if improvements from pre- to posttest were observed in the INT, the NCR might not be specific enough to give an impression of real-world orientation. For one, it is no real measure of large-scale spatial abilities, as the whole test set-up can be viewed from a single vantage point and does not require movement (Heil, 2020 based on Weatherford, 1982). Therefore, it does not provide sufficient spatial orientation and no spatial navigation information, which are essential for a complete picture of spatial abilities. Further, running speed and reaction time play essential roles for mastery of this task (Dirksen et al., 2015). Participant's motivation and state of mind on the testing day might additionally affect outcomes. Future research might make use of different, appropriate measures of spatial ability that can be used across age groups to evaluate whether theses capture intervention effects even more precisely. To date, virtual reality (VR) might be the most suitable approach to evaluate large-scale spatial abilities in a comparable, standardizable, and reproducible way. This however requires, that researchers have open access to testing material and measures and that they can access VR equipment (Uttal et al., 2024).

Even though no gender-specific differences were found in the present study, this factor should still be considered in future research. While sex differences in spatial abilities in favor of males have been discussed for many measures in adult populations (Linn and Petersen, 1985; Voyer et al., 1995), research in children is less conclusive. While some studies find sex differences in specific spatial abilities in pre-pubertal children and adolescents, others do not (e.g., Pavlovic, 2009; Neuburger et al., 2011; Blüchel et al., 2013; Jansen et al., 2013; Nazareth et al., 2019; Barhorst-Cates et al., 2020). Participant's age, task characteristics or solving strategies might play a critical role in the emergence of sex differences. It is further known that sex differences can be reduced to some degree by goal-oriented interventions (Feng et al., 2007; Tzuriel and Egozi, 2010). Even though some reasons are suspected (e.g., hormonal differences, exposure to spatial toys and games during childhood, cognitive developmental stages, usage of different strategies, or gender beliefs), it would be of great interest for future research to investigate the reasons for the development of sex difference in more detail (Linn and Petersen, 1985; Newhouse et al., 2007; Tzuriel and Egozi, 2010; Merrill et al., 2016; van der Heyden et al., 2016). This is of special interest with regard to academic achievements and occupational success, particularly in STEM subjects. Moreover, it would be of value to evaluate whether sex difference occur in the present study and whether the coordinative motor training addresses participants of all genders equally. Based on these findings, training programs could be tailored more specifically to facilitate spatial skills in all sexes and contribute to the reduction of sex differences.

Additionally, it can be discussed whether the implemented coordinative motor-training could be designed even more specifically to yield larger effects on spatial skills. The present intervention was built upon previous successful motor intervention studies (e.g., Blüchel et al., 2013; Jansen et al., 2013; Pietsch et al., 2017; Latino et al., 2021) and structured in such a way that the different sessions did not built upon each other and targeted different aspects of motor coordination. It was thus ensured that students could easily participate again at any time, even if they missed a session, which is essential within the school context. Moreover, the individual training sessions were designed with a focus on variety to keep participants motivated throughout the intervention period. Further, training content could be adapted to the participant's individual performance level in terms of complexity and degree of difficulty to forestall too high or too low loads. However, to date, little is known about the ideal training modalities (i.e., frequency, intensity, and duration) and content to maximize impact on spatial abilities. Previous research varies considerably and ranges from 20 min/session, 5x/week for 2 weeks to 40 min/session, 2x/week for 12 weeks (Blüchel et al., 2013; Latino et al., 2021). Future research should therefore consider to determine the most effective components of the present training to design more specific interventions. Beyond that, evaluating quantitative exercise parameters (e.g., heart rate, rating of perceived exertion, number of steps) might help to report exercise intensity more comprehensively in the future. Moreover, different training modalities should be evaluated and compared to find out how much training is required to yield significant effects and which training modalities yield the largest effects.

Building on this, it is further essential to establish how long-lasting intervention effects are. In the present study and most previous studies only the immediate post-intervention effects were evaluated. More research is needed to evaluate whether spatial skills continue to improve with ongoing training, whether a plateau is reached after some time as well as whether and how long intervention effects are maintained after the training is ceased.

## Conclusion

In conclusion it can be said that the 6-week coordinative motor training program with spatial elements performed in the present study has a positive impact on spatial abilities in healthy children. Thereby, results indicate that tailored physical training can be a suitable approach to foster cognitive functions and academic achievement, particularly in STEM-subjects. This strengthens the position of PE within the school context and at the same time helps to address various of the current health and academic challenges simultaneously. To establish a stronger and more detailed evidence base, more research on this topic is urgently needed.

## Data availability statement

The raw data supporting the conclusions of this article will be made available by the authors, without undue reservation.

## Ethics statement

The studies involving humans were approved by Human Ethics Committee of the University of Duisburg-Essen, Faculty of Educational Sciences. The studies were conducted in accordance with the local legislation and institutional requirements. Written informed consent for participation in this study was provided by the participants' legal guardians/next of kin.

## Author contributions

CM: Conceptualization, Data curation, Methodology, Writing—original draft, Writing—review & editing. AW: Data curation, Writing—review & editing. TM: Conceptualization, Data curation, Formal analysis, Methodology, Writing—review & editing.
